# Design, Synthesis and Biological Evaluation of Novel Pyrazolo[1,2,4]triazolopyrimidine Derivatives as Potential Anticancer Agents

**DOI:** 10.3390/molecules26134065

**Published:** 2021-07-02

**Authors:** Saeb Aliwaini, Bassam Abu Thaher, Ihab Al-Masri, Nabil Shurrab, Said El-Kurdi, Dieter Schollmeyer, Basem Qeshta, Mariam Ghunaim, René Csuk, Stefan Laufer, Lars Kaiser, Hans-Peter Deigner

**Affiliations:** 1 Department of Biology and Biotechnology, Islamic University of Gaza, Gaza P.O. Box 108, Palestine or saib.iwini@gmail.com (S.A.); mghunaim92@gmail.com (M.G.); 2 Chemistry Department, Faculty of Science, Islamic University of Gaza, Gaza P.O. Box 108, Palestine; skurdi@iugaza.edu.ps (S.E.-K.); basemsq@yahoo.com (B.Q.); 3 Faculty of Pharmacy, Al-Azhar University, Gaza P.O. Box 1277, Palestine; ihabalmasri@yahoo.com; 4 Chemistry Department, Al Azhar University-Gaza, Gaza P.O. Box 1277, Palestine; nabilkhsh139@yahoo.com; 5 Department of Organic Chemistry, Johannes Gutenberg-University Mainz, Duesbergweg 10-14, 55099 Mainz, Germany; scholli@uni-mainz.de; 6 Department of Organic Chemistry, Martin-Luther-University Halle-Wittenberg, Kurt-Mothes-Str. 2, 06120 Halle, Germany; rene.csuk@chemie.uni-halle.de; 7 Department of Pharmaceutical Chemistry, Pharmaceutical Institute, University of Tuebingen, Auf der Morgenstelle 8, 72076 Tuebingen, Germany; stefan.laufer@uni-tuebingen.de; 8 Institute of Precision Medicine, Faculty of Medical and Life Sciences, Furtwangen University (HFU), Jakob-Kienzle-Strasse 17, 78054 Villingen-Schwenningen, Germany; kal@hs-furtwangen.de; 9 Institute of Pharmaceutical Sciences, University of Freiburg, Albertstraße 25, 79104 Freiburg, Germany; 10 EXIM Department, Fraunhofer Institute IZI Leipzig, Schillingallee 68, 18057 Rostock, Germany; 11 Associated Member of Faculty of Science, Tuebingen University, Auf der Morgenstelle 8, 72076 Tübingen, Germany

**Keywords:** pyrazolo[1,2,4]triazolopyrimidine, EGF-receptor inhibitor, breast cancer, cervical cancer, molecular docking, crystal X-ray analysis

## Abstract

Three novel pyrazolo-[4,3-*e*][1,2,4]triazolopyrimidine derivatives (**1**, **2**, and **3**) were designed, synthesized, and evaluated for their in vitro biological activity. All three compounds exhibited different levels of cytotoxicity against cervical and breast cancer cell lines. However, compound **1** showed the best antiproliferative activity against all tested tumor cell lines, including HCC1937 and HeLa cells, which express high levels of wild-type epidermal growth factor receptor (EGFR). Western blot analyses demonstrated that compound **1** inhibited the activation of EGFR, protein kinase B (Akt), and extracellular signal-regulated kinase (Erk)1/2 in breast and cervical cancer cells at concentrations of 7 and 11 µM, respectively. The results from docking experiments with EGFR suggested the binding of compound **1** at the ATP binding site of EGFR. Furthermore, the crystal structure of compound **3** (7-(4-bromophenyl)-9-(pyridin-4-yl)-7*H*-pyrazolo[4,3-*e*][1,2,4]triazolo[1,5-*c*]pyrimidine) was determined by single crystal X-ray analysis. Our work represents a promising starting point for the development of a new series of compounds targeting EGFR.

## 1. Introduction

Cancer ranks in the top two of diseases (together with cardiovascular disease) with the highest death toll, and thus accounts for 13% of total deaths worldwide [1]. Breast and lung cancers are the most common cancers among women and men, respectively. Thus, enormous effort has been invested to address these diseases, but most current therapeutic strategies result in limited success. The strategy of targeting tyrosine kinase enzymes in human cancers has received increasing attention recently [2], since tyrosine kinases contribute to tumor initiation and progression by regulating cell growth, differentiation, migration, and apoptosis [3]. For these kinases, the epidermal growth factor receptor (EGFR) is the most important target for breast and lung cancer treatments, but it is also an attractive target for numerous other types of cancer (e.g., esophageal squamous cell carcinoma [4], colorectal cancer [5], head and neck squamous cell carcinoma [6], nasopharyngeal cancer [7], glioblastoma [8], and pancreatic cancer [9]). The activation of EGFR pathways activates cancer proliferation and increases metastasis potential, as well as neo-angiogenesis [10].

The inhibition of EGFR kinase activities by EGFR tyrosine kinase inhibitors (TKIs), however, is an effective treatment for patients with lung cancer [11]. Numerous studies conducted over the past decade have established that TKIs are therapeutically active in certain populations of cancer patients, and several of these drugs are components of current standard treatment regimens for specific cancers [12]. Importantly, treatment with a TKI is usually associated with longer progression-free survival and better response to treatment than standard chemotherapy [13]. One of the most clinically advanced strategies to offset abnormal EGFR receptor activation is the inhibition of the protein tyrosine kinase (TK) catalytic domain. Thereby, small-molecule TKIs compete with adenosine triphosphate (ATP) in the TK domain. Previous reports have shown that the kinase inhibitor binding site comprises five main pharmacophoric regions: a hydrophilic region formed of an adenine binding site, sugar and phosphate regions, and two hydrophobic areas, I and II [2]. First-generation TKIs (e.g., erlotinib, gefitinib, and lapatinib) are reversible small-molecule inhibitors that prevent the autophosphorylation of EGFR-TK by competing with ATP [10]. However, various EGFR mutations resulted in different sensitivities to these first-generation TKIs [14,15]. The second-generation TKIs (e.g., neratinib, pelitinib, afatinib, and dacomitinib), however, form covalent bonds with a conserved cysteine residue within the active site [15].

Another strategy developed to inhibit EGFR is the usage of monoclonal antibodies such as trastuzumab and cetuximab, which can control the growth of breast cancer cells overexpressing human epidermal growth factor receptor 2 (HER2). When trastuzumab attaches to HER2, it inhibits cell growth and leads to cell death [16]. Despite this success, resistance mechanisms have developed in many patients, thus limiting the use of these drugs [12]. To overcome these resistance mechanisms, further development has been made in the areas of immunotoxins composed of corresponding antibodies with a toxin fused to it, and of targeted toxins comprising a growth factor fused to the toxin [17,18].

The area of EGFR inhibitors, however, remains active and promising, and many articles have been published recently concerning the identification of effective inhibitors against mutant and/or wild-type receptors [13,19,20]. Moreover, great effort has been invested to discover irreversible EGFR inhibitors with new scaffolds [14,21]. Several computational techniques have been adopted for the discovery of new EGFR inhibitors, including virtual 3D-database screening, ligand-based pharmacophore modeling, and structure-based molecular modeling (e.g., molecular docking).

The heterocyclic pyrazolo[3,4-*d*]pyrimidine system has interesting chemical properties and a wide spectrum of biological activities [22]. New derivatives of pyrazolo[4,3-*e*][1,2,4] triazolo[1,5-*c*]pyrimidine act as kinase inhibitors and anticancer agents [22,23,24,25,26]. In a continuation of these efforts, we report the design, synthesis, and bioassay for pyrazolo-[4,3-*e*][1,2,4]triazolopyrimidine [1,2,3] and the crystal structure of 7-(4-bromophenyl)-9-(pyridin-4-yl)-7*H*-pyrazolo[4,3-*e*][1,2,4]triazolo[1,5-*c*]pyrimidine [3]. The synthesized compounds have been evaluated for antiproliferative activities against human tumor cells, their antitumor mechanisms, and effects on cell apoptosis, and cell cycle in vitro has been studied.

## 2. Results

### 2.1. Chemistry

A reaction of 4-pyridinecarboxaldehyde (I) with the corresponding arylhydrazines (II) afforded the corresponding hydrazones (III), as reported in the literature [26]. The chlorinating of the hydrazones (II) was performed with *N*-chlorosuccinimide (NCS) to yield hydrazonoyl chlorides (IV) [26]. The dried hydrazonoyl chlorides were reacted with dicyanomalonate in dry ethanol in the presence of sodium ethoxide (EtONa) to afford 5-aminopyrazolo-4-carbonitriles (V). These compounds (V) were reacted with ethyl orthoformate, affording formimidic esters (VI) according to the reported procedure [27]. As anticipated, the obtained compounds (VI) showed their correct molecular ions and their IR spectra revealed the existence of a CN group about 2230 cm^−1^ as it is appeared in the reactant (V), while the absence of NH_2_ band was recorded, which appears in the starting materials (V) around 3300–3100 cm^−1^, as well as the appearance of the vibrational band of C–O around 1250–1200 cm^−1^, confirmed the synthesized structure (VI).

All of these intermediates (I–VI) showed correct molecular ions, and the presence of the respective functional groups was confirmed by infrared (IR) spectra. Compounds VII and VIII were obtained via the reaction of compound VI with hydrazine hydrate for 24 h or 2 h, respectively [28]. Mass spectra (MS) for these compounds showed the correct quasimolecular ions, and their IR spectra revealed the presence of the functional groups, which definitely confirms that the reaction took place, where their IR spectra revealed the disappearance of the CN group which existed about 2230 cm^−1^ in reactant VI and again the appearance of NH_2_ and NH bands around 3300–3200 cm^−1^ that do not exist in VI.

**Scheme 1:**
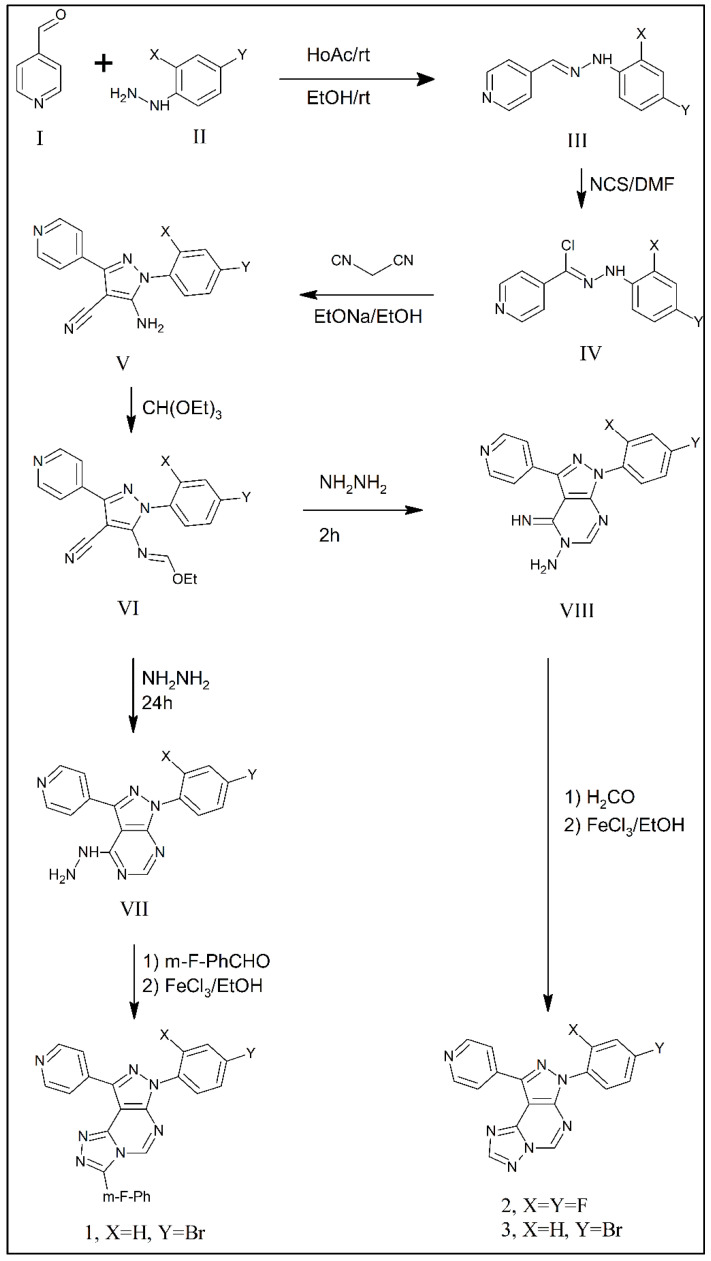
Synthesis of the target compounds **1**, **2**, and **3**.

**Figure 1:**
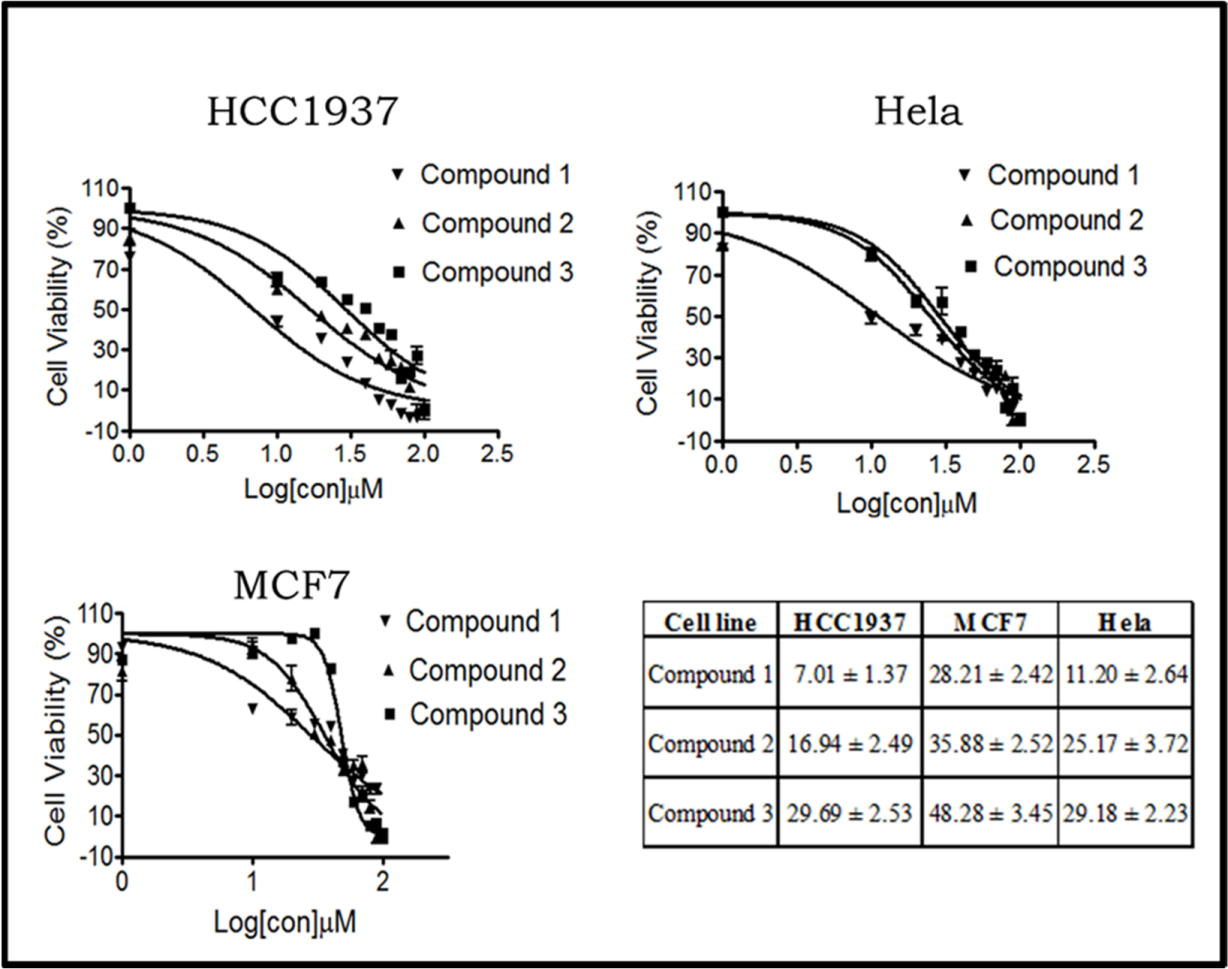
Cytotoxic effect of compounds **1**–**3** against breast (HCC1937 and MCF7) and cervical (HeLa) cancer cells. The cell lines were treated with increasing concentrations (1–100 μM) of compounds **1**–**3**. Cell viability was assessed by MTT assay after 48 h of treatment. The table shows IC_50_ values (mean ± SD) obtained from three independent determinations, each performed in quadruplicate. A control with solvent only (0.01% (*v*/*v*) DMSO) showed no effect on viability remaining at 100% (not shown).

Compounds VII and VIII were reacted with aromatic and/or aliphatic aldehydes to afford acyclic products. The target compounds pyrazolo[4,3-*e*][1,2,4]triazolo[1,5-*c*]pyrimidine **1**, **2**, and **3** were obtained by oxidative cyclization using FeCl_3_ (Scheme 1) [29,30]. The structures of the final products **1**–**3** were confirmed by IR, MS, and nuclear magnetic resonance (NMR) spectroscopy. Furthermore, compound **3** was assessed by single crystal X-ray analysis (Figure 1).

### 2.2. Biological Activity Evaluation

#### 2.2.1. Compounds **1**–**3** Show Different Cytotoxic Activities on Human Breast and Cervical Cancer Cells

The cytotoxic effects of the compounds **1**–**3** on both estrogen receptor (ER)-positive (MCF7) and ER-negative (HCC1937) breast cancer cell lines and on the HeLa cervical cancer cell line were examined employing MTT assays. The compounds inhibited the proliferation of cancer cells with IC_50_ values less than 50 µM (Figure 1). The most potent compound was compound **1**, and the most sensitive cells to all compounds were HCC1937 and HeLa cells. Importantly, both HCC1937 and HeLa cells express high levels of EGFR, suggesting an anti-EGFR mechanism of these compounds [31].

#### 2.2.2. Compound **1** Inhibits the EGFR/AKT Pathway in Both Breast and Cervical Cancer Cells

**Figure 2:**
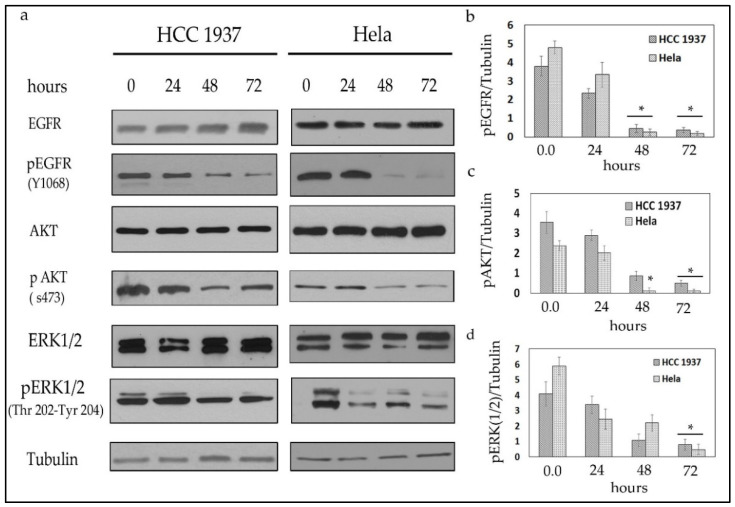
Compound **1** inhibits EGFR signaling in HCC1937 and HeLa cancer cells. (**a**) Western blot Table 1. at 7 and 11 µM for 24, 48 and 72 h and analyzed with antibodies to EGFR, pEGFR, AKT, pAKT, ERK1/2, and pERK1/2, respectively. Tubulin was used as a loading control. (**b**–**d**) Graphs representing densitometric quantitation of the magnitude of proteins in (**a**), the asterisk signifies statistical significance by one-sided Student’s *t*-test (* *p* < 0.05).

To test whether compound **1** cytotoxicity involved an inhibition of EGFR, Western blotting of key proteins from this pathway, including pEGFR, pAKT, and pERK1/2 [32] was performed. The results from these experiments demonstrated that treatment with compound **1** decreased pEGFR and its downstream targets, pAKT and pERK1/2, in both HCC1937 and HeLa cells (Figure 2). Although compound **1** treatment inhibited EGFR signaling in both cancer cell lines after 24 h, inhibition was more pronounced after 48 and 72 h of treatment. As a result, these findings indicate that compound **1** inhibits the EGFR pathway, regulating other important pathways such as cell cycle arrest and apoptosis.

#### 2.2.3. Compound **1** Induces Cell Cycle Arrest and Apoptosis in Both Breast and Cervical Cancer Cells

**Figure 3:**
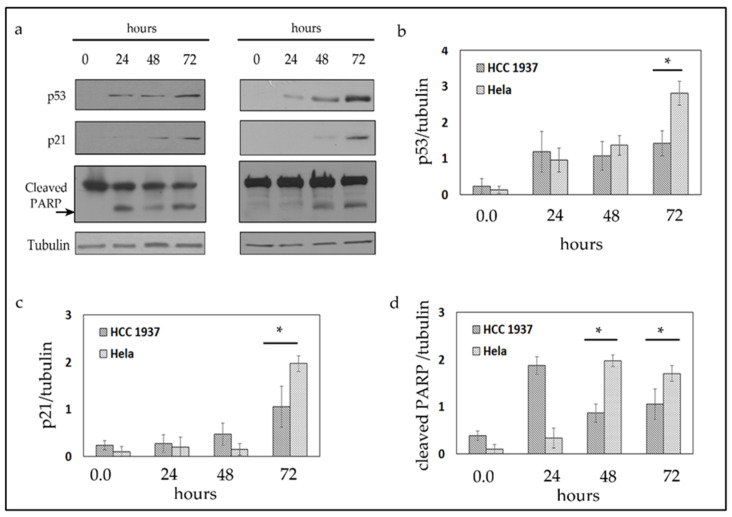
Compound **1** induces cell cycle arrest apoptosis in HCC1937 and HeLa cancer cells. (**a**) Western blots show protein from the indicated cancer cells treated as described in Figure 2 and analyzed with antibodies to p53, p21, and PARP. Tubulin was used as a loading control. (**b**–**d**) Graphs representing densitometric quantitation of the magnitude of proteins in (**a**), the asterisk signifies statistical significance by one-sided Student’s *t*-test (* *p* < 0.05).

To assess the possible effect of compound **1** on cell cycle arrest and apoptosis, HCC1937 and HeLa cancer cells were treated as described above, and Western blotting with markers of apoptosis and cell cycle arrest was performed. Figure 3 shows an obvious increase in p53 and p21 levels in both cancer cell lines after 48 h of treatment with compound **1**. Furthermore, cleaved poly (ADP-ribose) polymerase (PARP), an apoptotic marker, increased significantly after 48 h of treatment with compound 1 [33]. Consistent with these results, previous reports demonstrated that the inhibition of EGFR by erlotinib, gefitinib, cetuximab, or panitumumab leads to G1 cell cycle arrest and apoptosis [34,35]. These observations indicate that compound **1** inhibits EGFR signaling and induces both cell cycle arrest and apoptosis in HCC1937 and HeLa cells.

### 2.3. Molecular Docking Simulations

**Figure 4:**
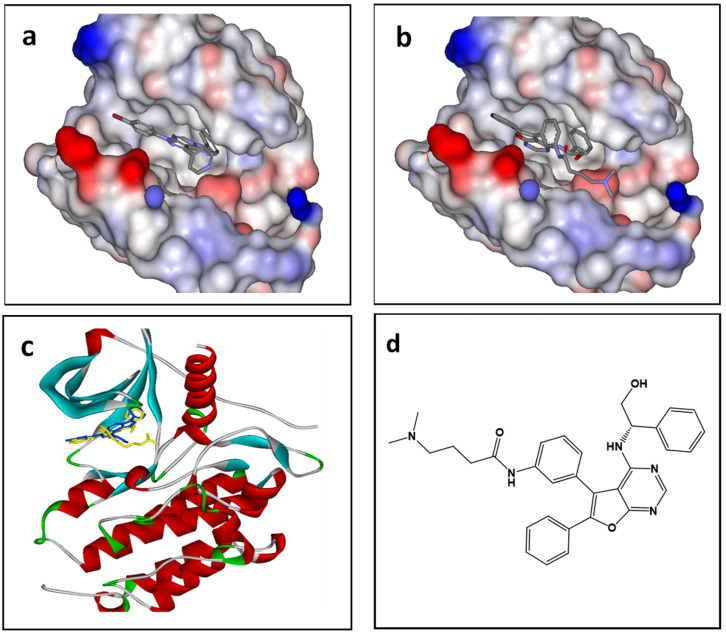
Solvent-accessible surface area of the active site of EGFR crystal structure 4JRV (Rs = 2.8 Å) containing the docked pose of pyrazoltriazolopyrimidine derivative **1** (**a**); and the co-crystallized inhibitor (furopyrimidine derivative) (**b**); ribbon diagram of docked pose of pyrazoltriazolopyrimidine derivative **1** (blue) superimposed on the co-crystallized ligand (yellow) (**c**); the structure of the furopyrimidine co-crystallized inhibitor (**d**).

**Figure 5:**
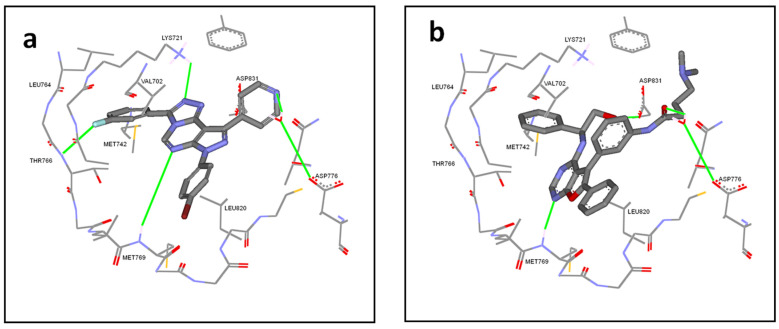
Detailed view of the docked poses of pyrazoltriazolopyrimidine derivative **1** (**a**) in the ATP binding site of EGFR showing hydrogen bonding with key amino acids compared with the co-crystallized ligand (**b**). Hydrogen bonds are indicated by green lines.

To reveal the binding features of the designed compounds with EGFR, we carried out molecular docking simulations on the synthesized compounds against the ATP binding site of EGFR using FRED software [36]. The performance of FRED was primarily validated on an EGFR crystal structure in complex with a furopyrimidine derivative, which demonstrated an improved EGFR inhibition, by the formation of a salt bridge with Asp831 in the DFG motif (Figure 4b; PDB code: 4JRV [37]). Re-docking (self-docking) of the co-crystallized ligand within the binding site of EGFR was carried out, and the obtained binding mode was examined. FRED successfully predicted the pose of the co-crystallized ligand with a lower root mean square deviation (RMSD) value. Subsequently, the docking of the synthesized compounds **1**–**3** was carried out, and the corresponding binding modes and binding energies were evaluated. Figure 4a shows the binding mode of the most active derivative (**1**), compared to the binding mode of the furopyrimidine derivative (Figure 4b–d) within the active site of EGFR. At the molecular level, the binding mode of derivative (**1**) in the active site reveals multiple favorable interactions with the active site residues of EGFR. Figure 5a shows that the pyrazolotriazolopyrimidine ring is hydrogen bonded to the hinge region between the amino and carboxy terminal lobes of the kinase receptor. The nitrogen atom of the pyrimidine ring in the tricyclic ring system is hydrogen bonded to the backbone amide NH of Met769, similar to the co-crystallized inhibitor (Figure 5b). Furthermore, the backbone NH of Thr766 and the terminal NH_2_ of Lys721 participate in a hydrogen-bonding network with fluorine in 3-fluorophenyl group and N_2_ of the triazole ring, respectively, whereas pyridine N makes a water-bridged hydrogen bond with Asp776 (Figure 5a), similar to the co-crystallized ligand. In contrast, the tricyclic ring is sandwiched between the side chains of Val702 and Leu820, with potential van der Waals interactions. The 3-fluorophenyl moiety is oriented deep in the hydrophobic pocket formed by the side chains of Lys721, Met742, Leu764, and Thr766, and makes predominantly hydrophobic interactions with the target protein (Figure 4a and Figure 5a). All of these potential interactions contribute to the formation of a tight ligand–EGFR complex with a total docking score of −12.61. On the other hand, compounds **2** and **3** had lower docking scores of −11.8 and −10.2, respectively.

### 2.4. Calculation of Molecular Properties and Drug-Likeliness

High oral bioavailability is often an essential requirement in the discovery and development of bioactive compounds as therapeutic agents. Thus, it is very important in drug design to consider the molecular properties that limit oral bioavailability to facilitate the design of feasible new drug candidates. Lipinski’s rule of five is commonly used in drug design and development to predict the oral bioavailability of potential lead compounds. The application of in silico computational tools throughout the drug discovery process to predict drug-likeliness characteristics saves time and money by reducing the number of experimental studies required for compound selection and development. Therefore, some important physicochemical parameters, for example, Lipinski’s parameters and polar surface area (PSA) of the synthesized compounds, were determined using the Molinspiration cheminformatics online software calculation toolkit. The absorption percentage (%ABS) was calculated by using the following formula: %ABS = 109 − 0.345 × PSA [38]. According to Lipinski’s rule of five, a potential lead will likely be orally active if the molecule satisfies the following: [38] (i) molecular weight (MW) ≤ 500, (ii) number of hydrogen bond donors (OH and NH groups) ≤ 5, (iii) number of hydrogen bond acceptors (notably N and O) ≤ 10, and (iv) calculated octanol/water partition coefficient (Log P) ≤ 5. Molecules violating more than one of these rules may cause problems with bioavailability (Table 1) [38].

The results in Table 1 revealed that the three designed compounds (**1**, **2**, and **3**) fulfill Lipinski’s rule of five without any violation. Two other descriptors were identified by Veber et al. (2002) [39]: the number of rotatable bonds (NRBs) ≤ 10 and PSA < 140 Å^2^. Compounds **1**, **2**, and **3** met the Lipinski and Veber rules of five, suggesting that these compounds theoretically have good oral bioavailability. Finally, the calculated percentage of absorption of all derivatives was 83.53%, indicating that they may have a good cell membrane permeability.

### 2.5. X-ray Crystallography

**Figure 6:**
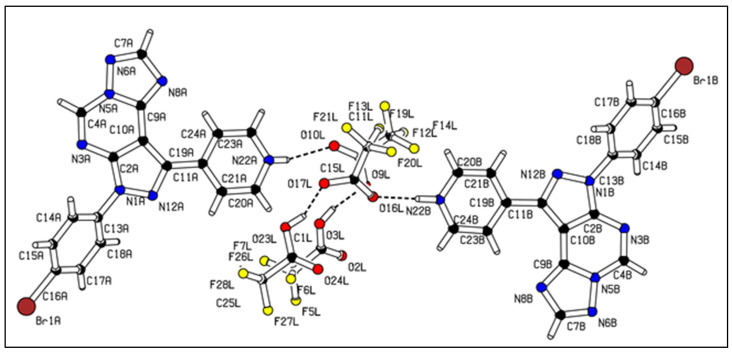
X-ray molecular structure of compound **3**.

The title compound, **3**, crystallizes from CF_3_COOH in the monoclinic space group P21/n. The structure contains two independent charged molecules A and B, two molecules CF_3_COOH/CF_3_COO^−^, and one solvent molecule, which could not be located. The values of the bond lengths and angles are given in Table S1 (Supplementary Materials). The molecular structure of the compound, with atom labeling, is shown in Figure 6. The bond lengths and angles in **3** are within normal ranges and comparable to those in the previously reported related derivatives of pyrazolotriazolopyrimidines [11,12,13,14,15,16,20].

In molecule **3**, the pyrazolotriazolopyrimidine ring system is almost planar; it forms dihedral angles of 45.1(3)° and 9.6(3)°, respectively, with the attached bromophenyl ring and pyridinyl fragment. Molecules A and B differ only in the conformation of the bromophenyl ring, which is rotated around 90° from A to B. The solvent, anion, and cation are linked via hydrogen bridges at N22A/B and on the solvent molecules (Figure 6).

In the pyrazolotriazolopyrimidine ring system, the C–N bonds are significantly shorter than a normal single C–N bond (1.47 Å) [9] and close to the value of a C=N bond (1.28 Å) [8]. This indicates a significant electron delocalization in the pyrazolotriazolopyrimidine system. The main characteristics of the H-bonds are listed in Table S2 (Supplementary Materials). The main intra- and intermolecular C–H···O and C–H···N hydrogen bonds contribute strongly to the stability of the crystal structure. The crystal structure motif is determined by the above-mentioned intermolecular interactions and can be characterized as a three-dimensional supramolecular network, as depicted in Figure S1 (Supplementary Materials).

## 3. Discussion

The synthesis of the intermediates VIIa and VIIIa,b begins by the reaction of *p*-pyridine car-boxaldehyde (I) with hydrazine derivatives (IIa,b) to produce the hydrazones (IIIa,b). IIIa,b were reacted with NCS to achieve the hydrazonoyl chloride (IVa,b). Compounds IVa,b were reacted with ethyl cyanomalonate to afford 5-aminopyrazolo-4-carbonitrile (Va,b) as reported in the literature [26]. Compounds Va,b were reacted with ethyl orthoformate affording formimidic ester (VIa,b), following the reported procedure [40]. The obtained compounds (VIa,b) showed the correct molecular ions and their IR spectra appeared in the related functional groups. Aminopyrazolopyrimidines VIIa and VIIIa,b were achieved via the reaction of compounds VIa,b with hydrazine hydrate according to literature procedure [27]. These compounds, VIIa and VIIIa,b, showed correct Ms and their IR spectra revealed the expected functional groups. Only Ms and IR were carried out for all the intermediates and then used without further purification for the next step. Compound VIIa was reacted with aromatic aldehydes to afford the acyclic product and then was oxidatively cyclized by using a commercial reagent FeCl_3_, affording the target compound **1**, while compounds VIIIa,b were reacted with formic acid under reflux, producing the other two target compounds **2** and **3**.

We designed and synthesized, through a multistep route, a series of novel pyrazolo-[4,3-*e*][1,2,4]triazolopyrimidine derivatives (**1**–**3**), the structures of which were confirmed by IR, MS, and NMR spectroscopy. In addition, suitable crystals of **3** were grown and subjected to a single crystal X-ray analysis as a representative example of this system.

This study is designed to prepare the two Regioisomers of this scaffold in order to explore the most effective one of them against some cancer cells in order to develop the system for the synthesis of more potency and efficiency inhibitors.

Compounds **1**–**3** exhibited distinct cytotoxicity against human breast and cervical cancer cells, with IC_50_ values ranging from 7.01 to 48.28 µM. Treatment with compound **1** inhibited the EGFR/AKT pathway and induced cell cycle arrest and apoptosis in both cancer cell types. Our study thus denotes a significant starting point to develop new EGFR-inhibiting drug candidates.

## 4. Materials and Methods

### 4.1. Computational Methodology (Molecular Modeling)

To explore the potential anticancer activity of the designed pyrazolotriazolopyrimidine derivatives (Scheme 1) against epidermal growth factor receptor (EGFR), molecular docking was used to reveal whether the proposed compounds could fit within the ATP binding site of the anticancer target. EGFR is a member of the ErbB family of receptors, which includes EGFR (ErbB-1), HER2/c-neu (ErbB-2), HER3 (ErbB-3), and HER4 (ErbB-4). Mutations affecting EGFR expression or activity have been associated with a number of cancers, including lung and anal cancer [41]. The selection of the target was based on structural similarity between the designed compound and some of the EGFR inhibitors and their structure–activity relationship requirements.

#### 4.1.1. Docking Experiment

The 2-dimensional chemical structure of the designed pyrazolotriazolopyrimidine derivatives (**1**–**3**; Scheme 1) were prepared in MarvinSketch [42] and saved in MDL molfile format. Subsequently, an ensemble of energetically accessible conformers was generated using OMEGA software [43]. OMEGA builds preliminary models of structures by assembling fragment templates along sigma bonds. The generated conformers were saved in SDF format. The X-ray crystal structure of EGFR (PDB ID: 4JRV) [37] was downloaded from the Protein Data Bank (www.rcsb.org (accessed on 14 May 2020)). Hydrogen atoms were added to proteins using Discovery Studio (DS) Visualizer templates for protein residues [44]. The receptor site for EGFR was identified using the pdb2receptor protocol within OEDocking package software [36]. The designed compounds were docked into the binding pocket of EGFR using FRED software. The ligand conformers and protein structure were treated as rigid during the docking process, which involved the exhaustive scoring of all possible positions of ligands in the active binding site [6]. The top scoring poses were optimized and assigned a final score.

#### 4.1.2. Calculation of Physicochemical Properties

The physicochemical and drug-likeliness properties of the designed compounds as potential leads were calculated using Molinspiration cheminformatics online software calculation toolkit, version 2018.10 (www.molinspiration.com (accessed on 10 August 2020)).

### 4.2. Chemistry

Silica gel (ADWIC 60 GF254) was used for thin layer chromatography (TLC). Column chromatography was performed on silica gel (70–230 mesh, Fluka). Melting points were obtained on a Gallenkamp melting point apparatus (open capillary tubes) (Weiss Gallenkamp, Loughborough, United Kingdom) and were uncorrected. NMR spectra were performed on a Bruker (400 MHz for ^1^H NMR and 100 MHz for ^13^C NMR) ultra-shield Avance III spectrometer (Bruker Corporation, Billerica, MA, United States), using Tetramethylsilane (TMS) as an internal standard and dimethyl sulfoxide (DMSO) or CDCl_3_/trifluoracetic acid (TFA) as solvents; chemical shifts were expressed as δ ppm. Electrospray ionization mass spectra (ESI) were performed on PE Sciex API Q-Star Pulsar Mass Spectrometer (AB Sciex LLC, Framingham, MA, United States). For accurate ion mass determinations, the MH^+^ ion was determined in high resolution.

#### General Procedure of the Synthesis of Pyrazolo[1,2,4]triazolopyrimidine Derivatives **1**, **2**, and **3**

To a solution of the appropriate hydrazone of compounds VII or VIII (5 mmol) in ethanol (125 mL), a solution of ferric chloride (2 M, 10 mL) was added; the mixture was stirred at room temperature overnight and the reaction was followed by TLC. The precipitate was filtered off, washed with water, ethanol, and petroleum ether, and finally crystallized from dimethylformamide (DMF) to give the respective pyrazolo-[4,3-*e*][1,2,4]triazolopyrimidine **1**, **2**, and **3**. (see Supplementary Materials Figures S2–S10 for corresponding IR, ^1^H and ^13^C NMR spectra).

*7-(4-Bromophenyl)-3-(3-fluorophenyl)-9-(pyridin-4-yl)-7H-pyrazolo[4,3-e][1,2,4]triazolo[4,3-c]pyrimidine* (**1**). White powder. Yield: 70%, M.P. = 336–337 °C, IR: 3060 (CH aromatic), 1637 (C=N), 1605, 1530, 1493 (benzene ring), 1478, 1449, 1427, 1416, 1406, 1387, 1359, 1322, 1304, 1278, 1262, 1204, 1075, 987, 824, 773. MS: 487–489 [M + H]^+^, 414, 340, 205. ^1^H NMR (400 MHz, CDCl3/TFA): δ 9.67 (d, *J* = 4.0 Hz, 2H), 9.57 (s, 1H), 9.01 (d, *J* = 4.0 Hz, 2H), 8.14 (d, *J* = 8.0 Hz, 2H), 8.03 (d, *J* = 8.0 Hz, 2H), 7.82 (d, *J* = 8.0 Hz, 2H) 7.60 (d, *J* = 8.0 Hz, 1H), 7.33 (br.s, 1H). ^13^C NMR (101 MHz, CDCl_3_/TFA): δ [165.18, 164.34 (d, 1C)], 149.09 (1C), 148.30 (1C), 147.49 (1C), 141.56 (1C), 139.79 (1C), 139.10 (1C), 136.16 (1C), 132.85 (1C), 131.02 (1C), 130.94 (1C), [129.98, 129.98 (d, 1C)], 124.69 (1C), 124.31 (1C), 123.59 (1C), 123.51 (1C), [119.18, 118.97 (1C, d)], [114.97, 114.97 (d, 1C)], 103.33 (1C).

*7-(2,4-Difluorophenyl)-9-(pyridin-4-yl)-7H-pyrazolo[4,3-e][1,2,4]triazolo[1,5-c]pyrimidine* (**2**). Pale yellow powder. Yield: 60%, M.P. = 279–280 °C, IR: 3049 (CH aromatic), 1634 (C=N), 1615, 1519 (benzene ring), 1426, 1387, 1349, 1325, 1304, 1272, 1253, 1206, 1151, 1112, 1063, 1031, 966, 843, 824, 777. MS: [M + H]^+^ = 350. ^1^H NMR (400 MHz, CDCl_3_/TFA): δ 9.55 (d, *J* = 6.3 Hz, 2H), 9.40 (s, 1H), 9.09 (d, *J* = 5.8 Hz, 2H), 8.63 (s, 1H), 7.79–7.71 (m, 1H), 7.24 (d, *J* = 3.5 Hz, 1H), 7.23–7.21 (m, 1H). ^13^C NMR (101 MHz, CDCl_3_/TFA): δ [165.19, 165.06–162.66, 162.55 (dd, 1C)], [158.47, 158.34–155.90, 155.78 (dd, 1C)], 155.05 (1C), 149.02 (1C), 148.27 (1C), 147.43 (1C), 142.04 (1C), 140.51 (1C), 140.33 (1C), [129.43 (d)], 124.54 (1C), [120.92, 120.87–120.79, 120.75 (dd, 1C)], [112.75, 112.72–112.53, 112.49 (dd, 1C)] [106.07, 105.84–105.81, 105.58 (dd, 1C)], 102.71 (1C).

*7-(4-Bromophenyl)-9-(pyridin-4-yl)-7H-pyrazolo[4,3-e][1,2,4]triazolo[1,5-c]pyrimidine* (**3**). Pale brown powder. Yield: 50%, M.P. = 263–264 °C, IR: 3064 (CH aromatic), 1684, 1637 (C=N), 1609, 1586, 1529, 1499 (benzene ring), 1354, 1324, 1306, 1257, 1226, 1216, 1175, 1151, 1071, 1013, 985, 826, 779. MS: 392–394 [M]^+^. ^1^H NMR (400 MHz, CDCl_3_/TFA): δ 9.59 (d, *J* = 6.5 Hz, 2H), 9.47 (s, 1H), 9.09 (d, *J* = 6.4 Hz, 2H), 8.66 (s, 1H), 8.18 (d, *J* = 8.7 Hz, 2H), 7.81 (d, *J* = 8.7 Hz, 2H). ^13^C NMR (101 MHz, CDCl_3_/TFA): δ 155.05 (1C), 148.35 (1C), 147.64 (1C), 147.39 (1C), 141.96 (1C), 140.01 (1C), 139.25 (1C), 136.42 (1C), 132.81 (1C), 124.57 (1C), 124.20 (1C), 123.16 (1C), 103.73 (1C).

### 4.3. X-ray Determination of Compound ***3***

Crystallographic measurements were carried out with IPDS 2T diffractometer (STOE & Cie GmbH, Darmstadt, Germany) equipped with graphite-monochromated Mo-Kα radiation. The single crystals were positioned 80 mm from the detector and 136 frames were measured each for 30 s over 1° scan width. The unit cell determination and the data integration were carried out using the X-Area software (STOE & Cie GmbH, Darmstadt, Germany). The structures were solved by direct methods using SHELXT-2014 software [45] and refined by the full-matrix least-squares method on F² with SHELXL-2018. Atomic displacements for non-hydrogen atoms were refined using an anisotropic model. Hydrogen atoms were placed in fixed, idealized positions and refined as rigidly bonded to the corresponding atoms. The crystal structure contains one more solvent molecule that was completely disordered, which was taken into account using the SQUEEZE procedure in PLATON [46]. The molecular plots were obtained using the PLATON program. The summary of the crystallographic data and the structure refinement are given in Table S3 (Supplementary Materials). CCDC 2042388 contains the crystallographic data for this contribution. These data can be obtained free of charge via www.ccdc.cam.ac.uk/conts/retrieving.html (or from the Cambridge Crystallographic Data Centre, 12 Union Road, Cambridge CB2 1EZ, UK; fax: (+44) 1223-336-033; or deposit@ccdc.ca.ac.uk).

### 4.4. Cell Culture and Treatments

Human triple-negative breast cancer cell line HCC1937, human breast adenocarcinoma (ER+) MCF7, and HeLa cervical cancer cells were maintained in RPMI 1640 medium (Biological Industries, Cat #01-106-1A). Medium was supplemented with 10% fetal bovine serum (FBS) and 1% penicillin–streptomycin solution (Biological Industries, Cat #03-031-1B). Cells were maintained at 37 °C in a 5% CO_2_–95% air humidified incubator. Compounds **1**, **2**, and **3** were dissolved in DMSO and used at different concentrations (1–100 µM). A vehicle control, consisting of DMSO only, was included as indicated in the figures, and the added volume of the tested compounds or the vehicle control did not exceed 0.1% (*v*/*v*).

### 4.5. MTT Assay for the Anti-Proliferative Activity In Vitro

To determine the cytotoxic effect of the indicated compounds, cancer cells were treated with compounds at a range of concentrations (1–100 µM) or vehicle (0.1% (*v*/*v*) DMSO) for 48 h. Cell viability was determined using the 3-(4,5-dimethylthiazol-2-yl)-2,5-diphenyltrazolium bromide (MTT) assay according to the manufacturer’s instructions (Santa Cruz, Cat #sc-359848A). Briefly, 10 μL of MTT solution was added to each well and incubated for 4 h at 37 °C. This was followed by the addition of 100 μL of solubilization buffer and overnight incubation at 37 °C. Absorbance at 585 nm was determined for each well and the mean cell viability was calculated as a percentage of the mean vehicle control by using an Automated ELISA Analyzer (CF-Fiocchetti, Luzzara, Italy). Three independent experiments were performed to determine the median inhibitory concentrations (IC_50_) of each compound. IC_50_ values were calculated by nonlinear regression based on sigmoidal dose–response (variable slope), using GraphPad Prism 5.0 (GraphPad Software Inc., USA).

### 4.6. Western Blotting

Cells were harvested and protein prepared as described previously [33]. The primary antibodies used were anti-EGFR, anti-pEGFR, anti-AKT, anti-pAKT, anti-PARP1/2 (sc-7150), anti-p53 (sc-126), anti-p21 (sc-756), and anti-α-tubulin (sc-8035) (Santa Cruz Biotechnology, Santa Cruz, CA, USA), anti-phospho-p44/42 MAP kinase (#9101) (Cell Signaling, Boston, MA, USA).

## Figures and Tables

**Table 1:** Drug-likeliness prediction of the synthesized compounds using Molinspiration v2018.10. PSA: polar surface area; NRB: number of rotatable bonds; miLogP: Log partition coefficient; HBA: number of hydrogen bond acceptors; HBD: number of hydrogen bond donors; Mol Vol: molecular volume; MW: molecular weight; %ABS: percentage absorption.

Compound	PSA	NRB	miLogP	HBA	HBD	Mol Vol	MW	%ABS
**1**	73.81	3	4.44	7	0	359.02	486.31	83.53%
**2**	73.81	2	1.84	7	0	274.66	349.30	83.53%
**3**	73.81	2	2.19	7	0	282.68	392.22	83.53%

## Data Availability

The data presented in this study can be obtained free of charge via www.ccdc.cam.ac.uk/conts/retrieving.html (or from the Cambridge Crystallographic Data Centre, 12 Union Road, Cambridge CB2 1EZ, UK; fax: (+44) 1223-336-033; or deposit@ccdc.ca.ac.uk).

## Supplementary Materials

The following are available online at, Table S1: selected bond lengths and angles geometric parameters (Å, °), Table S2: hydrogen bond geometry (Å, °), Table S3: crystallographic data, details of data collection and structure refinement parameters for compound **3**, Figure S1: three-dimensional supramolecular network derived from intramolecular interactions of compound **3**. Figures S2 to S10: corresponding IR, ^1^H and ^13^C NMR spectra of compounds **1** to **3**.

## Sample Availability

Samples of the compounds **1**, **2** and **3** are available from the author B.A.T. upon reasonable request.
