# Supplementary material for: Design, Synthesis and Biological Evaluation of Novel Pyrazolo[1,2,4]triazolopyrimidine Derivatives as Potential Anticancer Agents
Source: Molecules. 2021 Jul 2;26(13):4065. doi: 10.3390/molecules26134065 (PMC8271544; doi:10.3390/molecules26134065)
Supplement: Supplementary file 1 [file molecules-26-04065-s001.zip › molecules-1268264-supplementary.pdf]

Supplementary data

for

Design, Synthesis and Biological Evaluation of Novel Pyrazolo[1,2,4]triazolopyrimidine Derivatives as Potential Anticancer Agents

Saeb Aliwaini [1], Bassam Abu Thaher [2,*], Ihab Al-Masri [3], Nabil Shurrab [4], Said El-Kurdi [2], Dieter Schollmeyer [5], Basem Qeshta [2], Mariam Ghunaim [1], René Csuk [6], Stefan Laufer [7], Lars Kaiser [8,9] and Hans-Peter Deigner [8,10,11,*]

[1] Department of Biology and Biotechnology, Islamic University of Gaza, Gaza PO Box 108, Palestine; siwini@iugaza.edu.gs (S.A.); mghunaim92@gmail.com (M.G.)
[2] Faculty of Science, Chemistry Department, Islamic University of Gaza, Gaza P.O. Box 108, Palestine; skurdi@iugaza.edu.ps (S.E.-K.); basemsq@yahoo.com (B.Q.)
[3] Faculty of Pharmacy, Al-Azhar University, Palestine; ihabalmasri@yahoo.com
[4] Chemistry Department, Al Azhar University-Gaza, Gaza, Palestine; nabilkhsh139@yahoo.com
[5] Department of Organic Chemistry, Johannes Gutenberg-University Mainz, Duesbergweg 10-14, 55099 Mainz, Germany; scholli@uni-mainz.de
[6] Department of Organic Chemistry, Martin-Luther-University Halle-Wittenberg, Kurt-Mothes-Str. 2, 06120 Halle (Saale), Germany; rene.csuk@chemie.uni-halle.de
[7] Department of Pharmaceutical Chemistry, Pharmaceutical Institute, University of Tuebingen, Auf der Morgenstelle 8, 72076 Tuebingen, Germany; stefan.laufer@uni-tuebingen.de
[8] Faculty of Medical and Life Sciences, Institute of Precision Medicine, Furtwangen University (HFU), Jakob-Kienzle-Strasse 17, 78054 Villingen-Schwenningen, Germany; kal@hs-furtwangen.de
[9] Institute of Pharmaceutical Sciences, University of Freiburg, Albertstraße 25, 79104 Freiburg i. Br., Germany
[10] EXIM Department, Fraunhofer Institute IZI Leipzig, Schillingallee 68, 18057 Rostock, Germany
[11] Associated member of Faculty of Science, Tuebingen University, Auf der Morgenstelle 8, 72076 Tübingen, Germany
* Correspondence: Hans-Peter.Deigner@hs-furtwangen.de (H.-P.D.); bthaher@iugaza.edu.ps (B.A.T.); Tel.: +49-7720-307-4232 (H.-P.D.); +970-8-264-4400 (B.A.T)

Table S1. Selected bond lengths and angles Geometric parameters (Å, º)

Br1A—C16A	1.909(7)	C7B—H7B	0.9500
N1A—C2A	1.355(8)	N8B—C9B	1.314(8)
N1A—N12A	1.355(7)	C9B—C10B	1.435(9)
N1A—C13A	1.437(8)	C10B—C11B	1.412(9)
C2A—N3A	1.374(8)	C11B—N12B	1.335(8)
C2A—C10A	1.383(8)	C11B—C19B	1.463(9)
N3A—C4A	1.292(8)	C13B—C14B	1.380(9)
C4A—N5A	1.377(8)	C13B—C18B	1.388(9)
C4A—H4A	0.9500	C14B—C15B	1.379(9)
N5A—C9A	1.363(8)	C14B—H14B	0.9500
N5A—N6A	1.386(8)	C15B—C16B	1.385(9)
N6A—C7A	1.289(9)	C15B—H15B	0.9500
C7A—N8A	1.370(9)	C16B—C17B	1.366(9)
C7A—H7A	0.9500	C17B—C18B	1.392(9)
N8A—C9A	1.321(8)	C17B—H17B	0.9500
C9A—C10A	1.431(9)	C18B—H18B	0.9500
C10A—C11A	1.418(8)	C19B—C20B	1.389(9)
C11A—N12A	1.329(8)	C19B—C24B	1.394(9)
C11A—C19A	1.464(9)	C20B—C21B	1.351(10)
C13A—C18A	1.381(9)	C20B—H20B	0.9500
C13A—C14A	1.402(9)	C21B—N22B	1.336(9)
C14A—C15A	1.375(9)	C21B—H21B	0.9500
C14A—H14A	0.9500	N22B—C23B	1.338(9)
C15A—C16A	1.392(10)	N22B—H22B	0.92(8)
C2A—N1A—N12A	111.3(5)	N8B—C9B—C10B	135.2(6)
C2A—N1A—C13A	129.0(5)	N5B—C9B—C10B	114.4(6)

N12A—N1A—C13A	119.7(5)	C2B—C10B—C11B	105.6(5)
N1A—C2A—N3A	124.0(6)	C2B—C10B—C9B	115.1(6)
N1A—C2A—C10A	107.2(5)	C11B—C10B—C9B	139.0(6)
N3A—C2A—C10A	128.8(6)	N12B—C11B—C10B	109.9(5)
C4A—N3A—C2A	114.0(6)	N12B—C11B—C19B	117.7(6)
N3A—C4A—N5A	121.7(6)	C10B—C11B—C19B	132.4(6)
N3A—C4A—H4A	119.100	C11B—N12B—N1B	106.5(5)
N5A—C4A—H4A	119.100	C14B—C13B—C18B	121.1(6)
C9A—N5A—C4A	126.1(6)	C14B—C13B—N1B	121.2(6)
C9A—N5A—N6A	108.6(5)	C18B—C13B—N1B	117.6(6)
C4A—N5A—N6A	125.4(6)	C15B—C14B—C13B	119.8(6)
C7A—N6A—N5A	102.0(6)	C15B—C14B—H14B	120.100
N6A—C7A—N8A	117.1(7)	C13B—C14B—H14B	120.100
N6A—C7A—H7A	121.400	C14B—C15B—C16B	119.0(6)
N8A—C7A—H7A	121.400	C14B—C15B—H15B	120.500
C9A—N8A—C7A	101.8(6)	C16B—C15B—H15B	120.500
N8A—C9A—N5A	110.5(6)	C17B—C16B—C15B	121.7(6)

N8A—C9A—C10A	135.2(6)	C17B—C16B—Br1B	119.8(5)
N5A—C9A—C10A	114.3(6)	C15B—C16B—Br1B	118.5(5)
C2A—C10A—C11A	104.8(5)	C16B—C17B—C18B	119.6(6)
C2A—C10A—C9A	115.1(5)	C16B—C17B—H17B	120.200
C11A—C10A—C9A	140.1(6)	C18B—C17B—H17B	120.200
N12A—C11A—C10A	110.4(5)	C13B—C18B—C17B	118.9(6)
N1B—C2B—N3B	124.1(6)	F20L—C18L—F21L	106.8(8)
C10B—C2B—N3B	128.8(6)	F19L—C18L—F21L	104.7(8)
C4B—N3B—C2B	114.5(6)	F20L—C18L—C15L	110.9(8)
N3B—C4B—N5B	121.6(6)	F19L—C18L—C15L	110.9(7)
N3B—C4B—H4B	119.200	F21L—C18L—C15L	115.2(7)
N5B—C4B—H4B	119.200	O24L—C22L—O23L	127.5(9)
C4B—N5B—N6B	125.9(6)	O24L—C22L—C25L	119.2(9)
C4B—N5B—C9B	125.6(6)	O23L—C22L—C25L	113.3(8)
N6B—N5B—C9B	108.5(6)	C22L—O23L—H23L	109.500
C7B—N6B—N5B	101.3(6)	F27L—C25L—F28L	111.3(10)
N12A—N1A—C2A—N3A	176.9(6)	N6B—C7B—N8B—C9B	-0.6(9)

C13A—N1A—C2A—N3A	-0.9(11)	C7B—N8B—C9B—N5B	1.3(7)
N12A—N1A—C2A—C10A	-1.6(7)	C7B—N8B—C9B—C10B	-179.3(8)
C13A—N1A—C2A—C10A	-179.4(6)	C4B—N5B—C9B—N8B	179.4(6)
N1A—C2A—N3A—C4A	-177.9(6)	N6B—N5B—C9B—N8B	-1.5(8)
C10A—C2A—N3A—C4A	0.3(10)	C4B—N5B—C9B—C10B	-0.2(9)
C2A—N3A—C4A—N5A	-1.4(9)	N6B—N5B—C9B—C10B	178.9(6)
N3A—C4A—N5A—C9A	1.4(11)	N1B—C2B—C10B—C11B	-1.9(7)
N3A—C4A—N5A—N6A	-179.7(6)	N3B—C2B—C10B—C11B	175.1(6)

Table S2. Hydrogen-bond geometry (Å, º)

D-H...A	d(D-H)	d(H...A)	d(D...A)	<(DHA)
C(4A)-H(4A)...O(10L)#1	0.95	2.40	3.200(8)	142.1
C(4A)-H(4A)...F(13L)#1	0.95	2.43	3.174(8)	134.8
C(14A)-H(14A)...O(17L)#1	0.95	2.60	3.397(9)	142.2
C(15A)-H(15A)...O(23L)#1	0.95	2.59	3.462(9)	152.4
C(21A)-H(21A)...O(3L)	0.95	2.55	3.230(9)	129.1
C(21A)-H(21A)...O(23L)	0.95	2.60	3.135(10)	116.2
N(22A)-H(22A)...O(10L)	0.78	2.02	2.743(7)	154.8
C(23A)-H(23A)...O(2L)#2	0.95	2.51	3.354(9)	148.4
C(24A)-H(24A)...N(8A)	0.95	2.30	3.177(9)	153.5
C(4B)-H(4B)...O(24L)#3	0.95	2.64	3.489(10)	149.7
C(15B)-H(15B)...F(14L)#3	0.95	2.56	3.316(8)	136.3
C(17B)-H(17B)...O(2L)#4	0.95	2.52	3.455(9)	169.4
N(22B)-H(22B)...O(16L)	0.92	1.84	2.729(8)	160.3
C(23B)-H(23B)...N(3B)#5	0.95	2.61	3.389(9)	139.3
C(24B)-H(24B)...N(8B)	0.95	2.34	3.221(9)	154.0
O(3L)-H(3L)...O(9L)	0.84	1.64	2.435(8)	156.2
O(23L)-H(23L)...O(17L)	0.84	1.65	2.425(9)	151.5

Symmetry codes:

#1 x-1/2,-y+1/2,z-1/2 #2 -x+1,-y+1,-z+1
#3 x+1/2,-y+3/2,z+1/2 #4 x+1,y,z+1
#5 x-1/2,-y+3/2,z-1/2

Table S3. Crystallographic data, details of data collection and structure refinement parameters for compound **3**.

formula	$C_{17}H_{11}BrN_7^+$, CF_3COOH, CF_3COO^- [+ solvent]		
molecular weight	620.29 $gmol^{-1}$		
absorption	μ = 1.72 mm^{-1}		
crystal size	0.11 x 0.14 x 0.17 mm^3 brown block		
space group	P 2_1/n (monoclinic)		
lattice parameters	a = 14.6974(4)Å		
(calculate from	b = 25.0001(9)Å	ß = 115.489(2)°	
12243 reflections with	c = 15.1766(5)Å		
2.56° <θ< 28.17°)	V = 5033.7(3)$Å^3$	z = 8	F(000) = 2464
temperature	-80°C		
density	d_{xray} = 1.637 gcm^{-3}		

data collection

diffractometer	STOE IPDS 2T
radiation	Mo-K_α Graphitmonochromator
Scan – type	ω scans
Scan – width	1°
scan range	2° ≤ θ< 28°
	-19 ≤ h ≤ 19 -33 ≤ k ≤ 28 -20 ≤ l ≤ 20
number of reflections:	
measured	26865
unique	12502 (R_{int} = 0.0568)
observed	6103 (|F|/σ(F) > 4.0)

data correction, structure solution and refinement

corrections	Lorentz and polarisation correction.
Structure solution	Program: SHELXT-2014
refinement	Program: SHELXL-2018 (full matrix). 705 refined parameters, weighting scheme: $w=1/[\sigma^2(F_o^2) + (0.0767*P)^2+20.39*P]$ with $(Max(F_o^2,0)+2*F_c^2)/3$. H-atoms at calculated positions and refined with isotropic displacement parameters, NH's and OH's localized, non H- atoms refined anisotropically.
R-values	wR2 = 0.2387 (R1 = 0.0851 for observed reflections, 0.2387 for all reflections)
goodness of fit	S = 1.019
maximum deviation of parameters	0.001 * e.s.d

maximum peak height in diff. Fourier synthesis	0.61, -0.49 eÅ^{-3}
Remark	crystal structure contains a void filled with solvent which could not be located → SQUEEZE was used

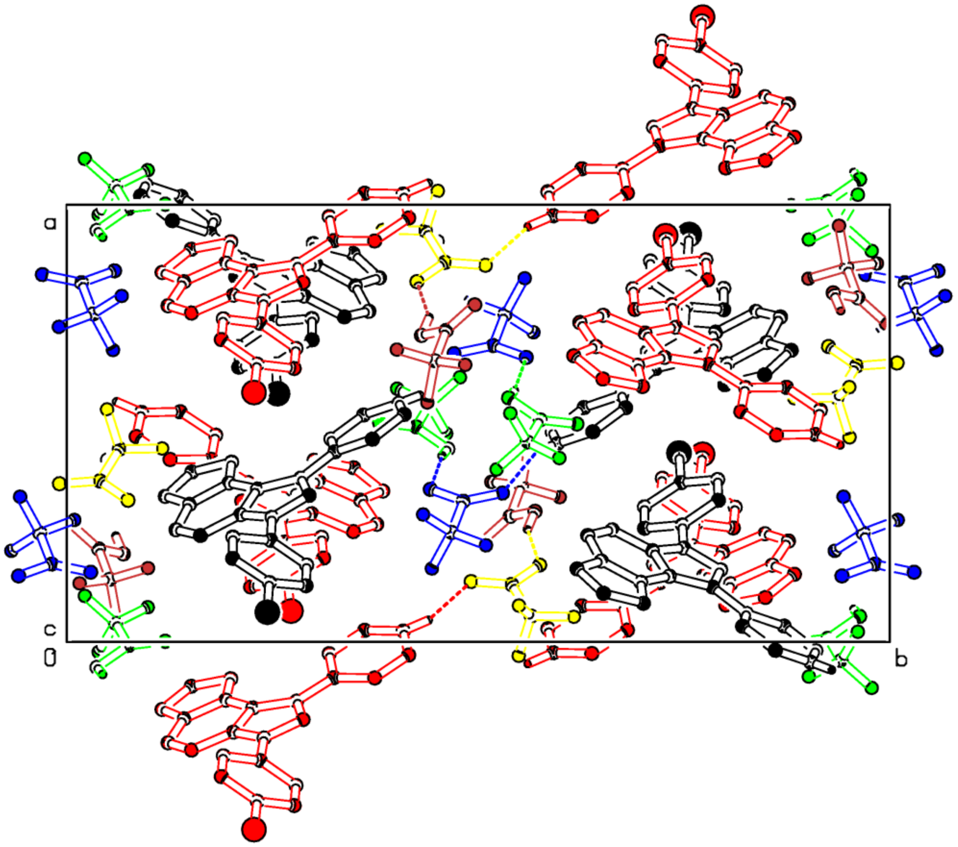

Figure S1. Three-dimensional supramolecular network derived from intramolecular interactions of compound **3.**

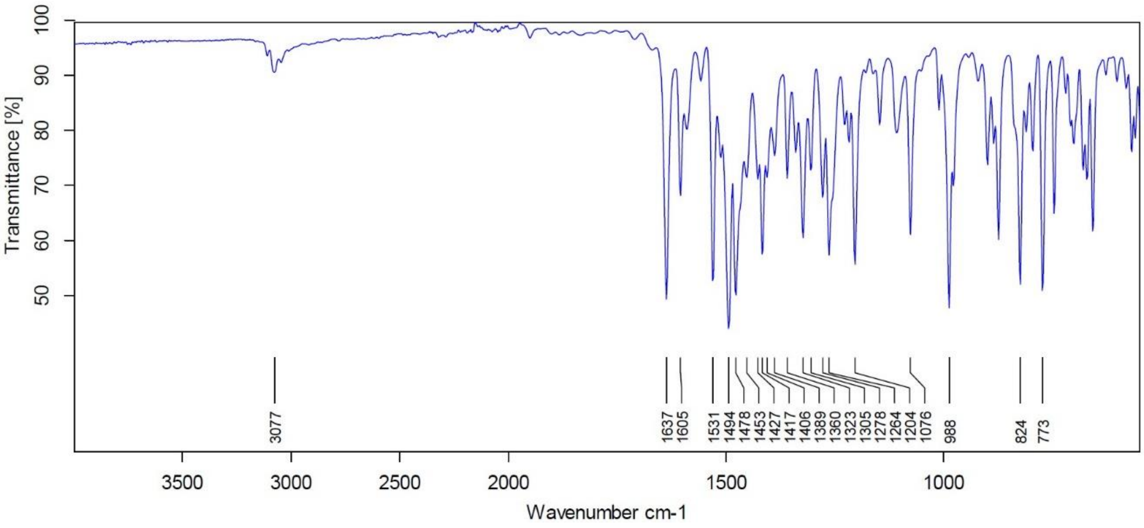

Figure S2. IR spectrum of compound 1.

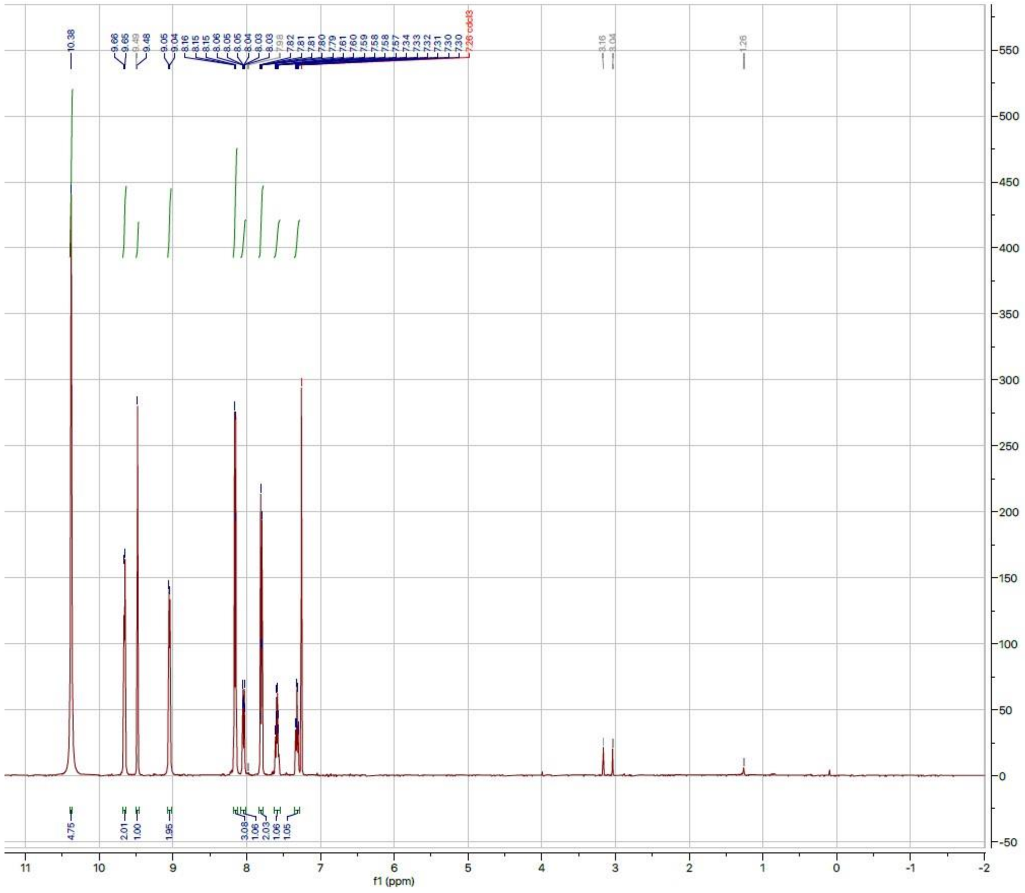

Figure S3. 1H NMR spectrum of compound 1.

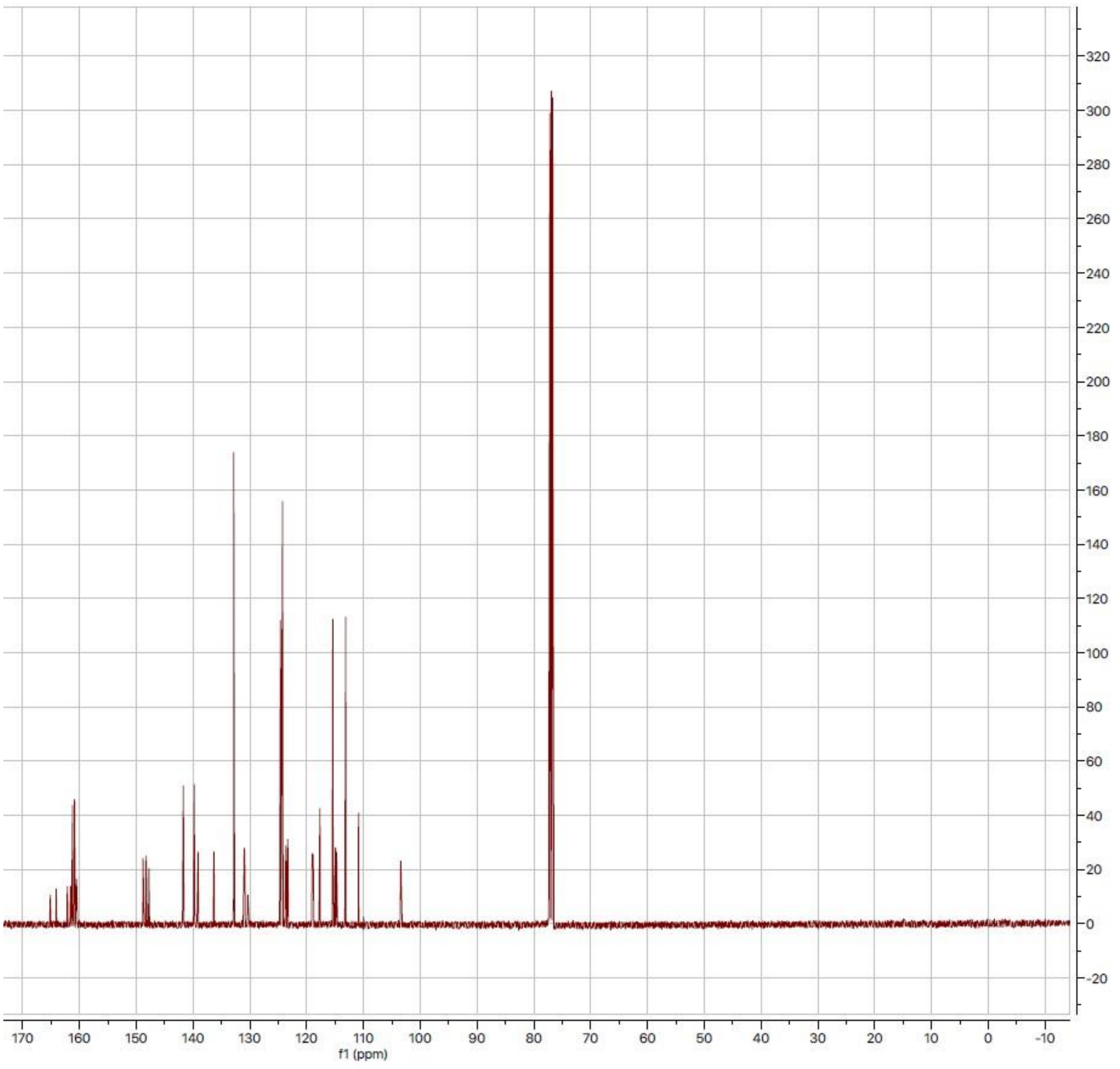

Figure S4. 13C NMR spectrum of compound 1.

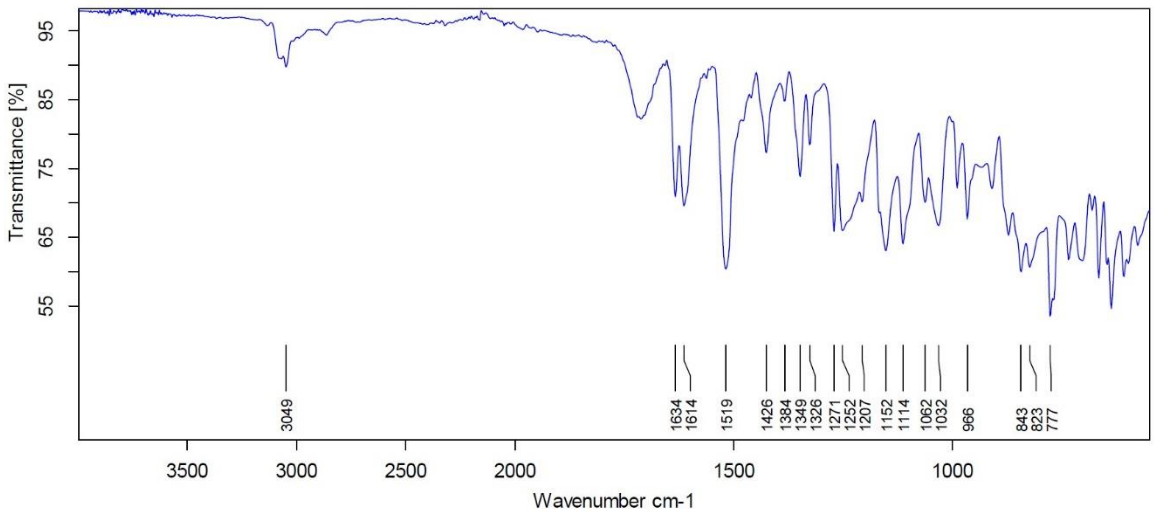

Figure S5. IR spectrum of compound 2.

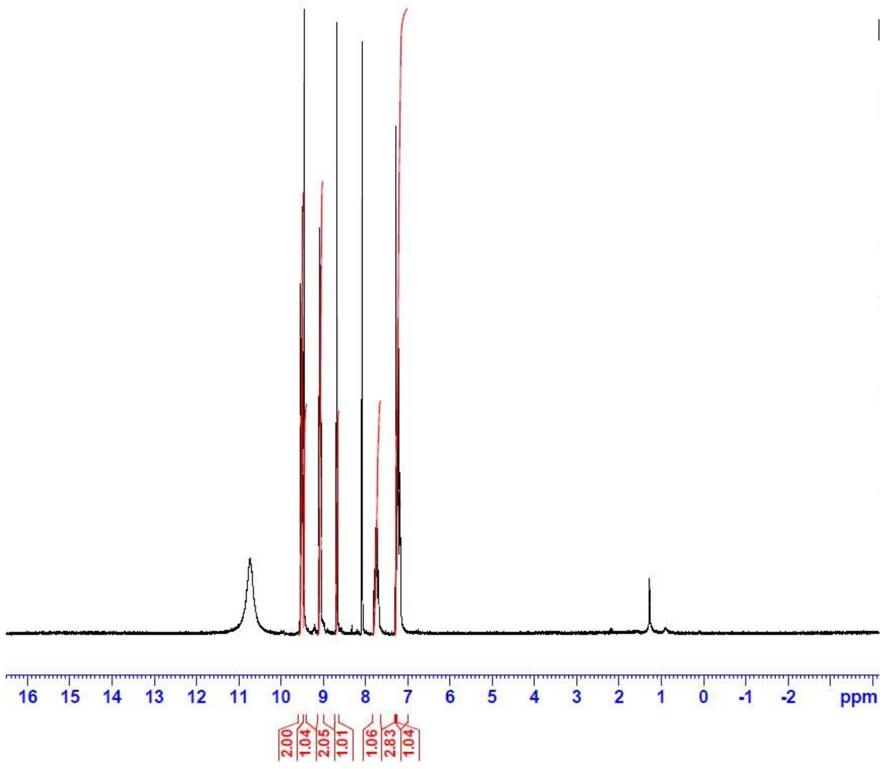

Figure S6. 1H NMR spectrum of compound 2.

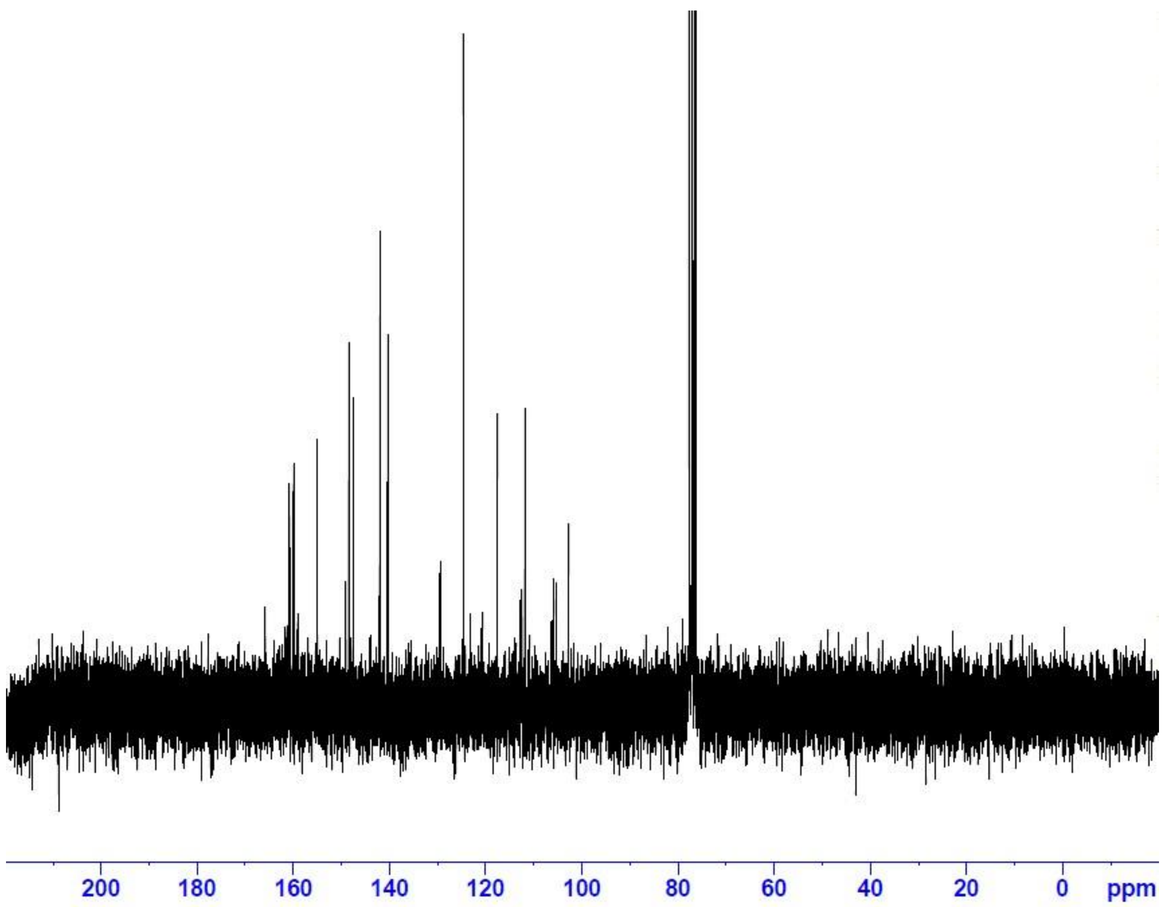

Figure S7. 13C NMR spectrum of compound 2.

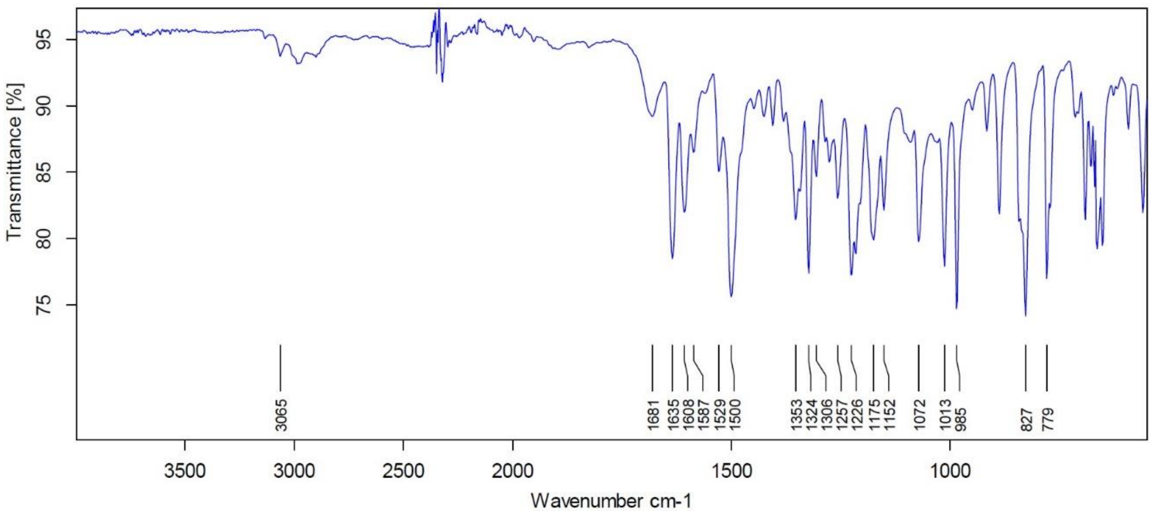

Figure S8. IR spectrum of compound 3.

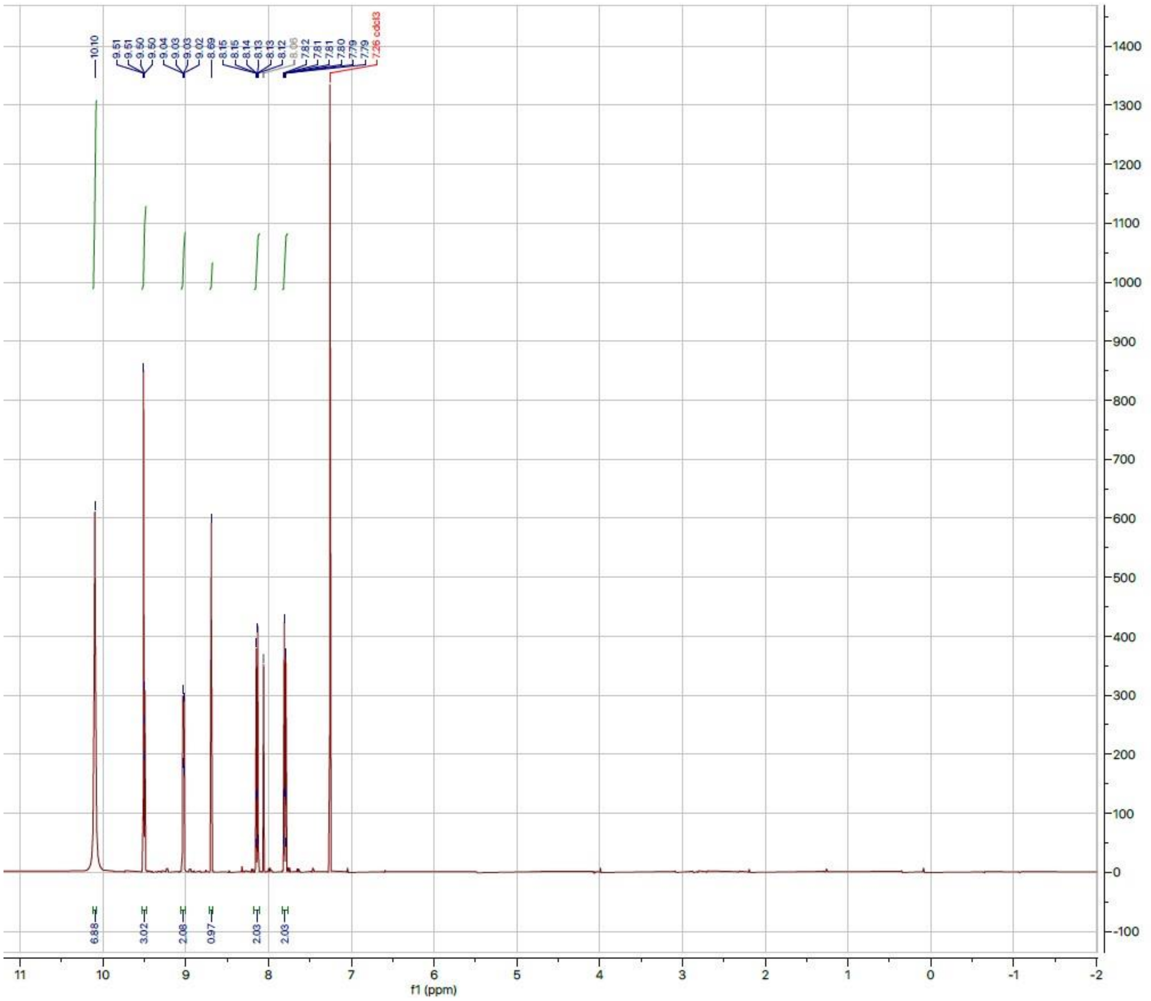

Figure S9. 1H NMR spectrum of compound 3.

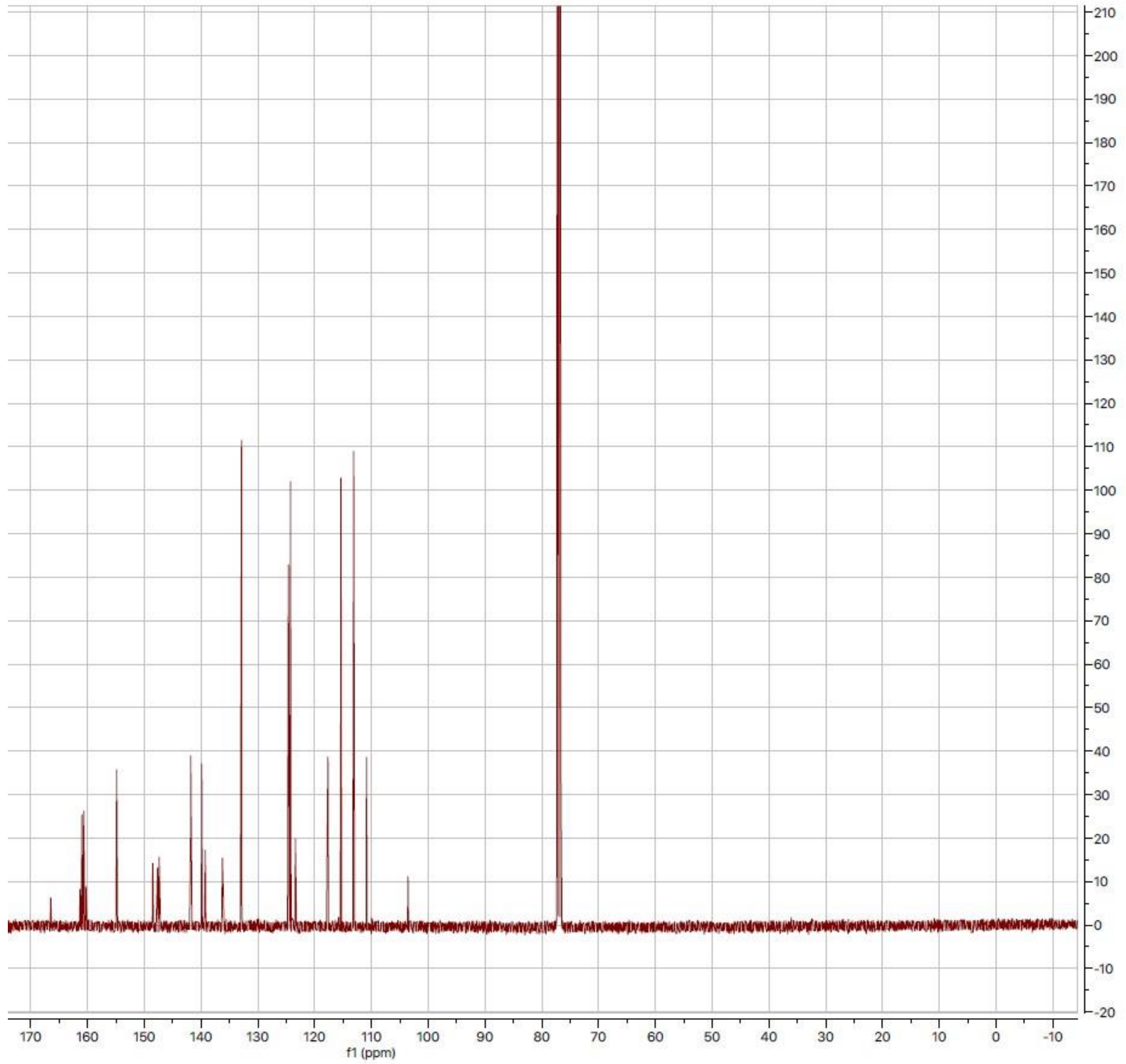

Figure S10. 13C NMR spectrum of compound 3.
